# Socioeconomic inequalities in the prevalence of underweight, overweight, and obesity among women aged 20–49 in low- and middle-income countries

**DOI:** 10.1038/s41366-019-0503-0

**Published:** 2019-12-18

**Authors:** Ursula Reyes Matos, Marilia Arndt Mesenburg, Cesar G. Victora

**Affiliations:** International Center for Equity in Health, Postgraduate Program in Epidemiology, Federal University of Pelotas, Marechal Deodoro 1160, Pelotas, Rio Grande do Sul Brazil

**Keywords:** Nutrition, Epidemiology, Epidemiology

## Abstract

**Objective:**

To analyze socioeconomic inequalities in the prevalence of underweight and overweight or obesity in women from low and middle-income countries (LMICs).

**Methods:**

Using the last available Demographic Health Survey between 2010 and 2016 from 49 LMICs, we estimated the prevalence of underweight (BMI < 18.5 kg/m^2^) and overweight or obesity combined (BMI ≥ 25 kg/m^2^) for women aged 20–49 years. We used linear regression to explore the associations between the two outcomes and gross national income (GNI). We assess within-country socioeconomic inequalities using wealth deciles. The slope index of inequality (SII) and the inequality pattern index (IPI) were calculated for each outcome. Negative values of the latter express bottom inequality (when inequality is driven by the poorest deciles) while positive values express top inequality (driven by the richest deciles).

**Results:**

In total, 931,145 women were studied. The median prevalence of underweight, overweight or obesity combined, and obesity were 7.3% (range 0.2–20.5%), 31.5% (8.8–85.3%), and 10.2% (1.9–48.8%), respectively. Pearson correlation coefficients with log GNI were −0.33 (*p* = 0.006) for underweight, 0.72 (*p* < 0.001) for overweight or obesity, and 0.66 (*p* < 0.001) for obesity. For underweight, the SII was significantly negative in 38 of the 49 countries indicating a higher burden among poor women. There was no evidence of top or bottom inequality. Overweight or obesity increased significantly with wealth in 44 of the 49 countries. Top inequality was observed in low-prevalence countries, and bottom inequality in high-prevalence countries.

**Conclusion:**

Underweight remains a problem among the poorest women in poor countries, but overweight and obesity are the prevailing problem as national income increases. In low-prevalence countries, overweight or obesity levels are driven by the higher prevalence among the richest women; as national prevalence increases, only the poorest women are relatively preserved from the epidemic.

## Introduction

The spectrum of malnutrition includes both undernutrition (for example, low body mass index or BMI) and overweight or obesity [1]. Our analyses address how the prevalence of the conditions representing these two extremes of the malnutrition spectrum vary according to socioeconomic position in women living in low- and middle-income countries (LMICs).

According to global estimates, underweight affects around 462 million adults [1], representing a serious problem among reproductive age women for their own health and for the health and nutrition of their offspring. Low pre-gestational BMI is an important determinant of adverse newborn and child outcomes, such as preterm birth, low-birth weight, under-five mortality, and of poor mental and physical development [2].

In addition to the persisting problem of underweight, many LMICs are showing the increasing prevalence of overweight or obesity among adults, similar to what is observed in high-income countries [3]. In 2016, the World Health Organization estimated that 1.9 billion adults (or 39% of the total population) presented overweight or obesity, of whom 650 million (or 13% of the total) were obese [4]. Overweight or obesity are major risk factors for noncommunicable diseases such as diabetes, musculoskeletal disorders, some cancers (including breast, ovarian and liver) and cardiovascular diseases, the leading causes among women [4]. Maternal overweight or obesity also contribute to adverse obstetric and neonatal outcomes such as congenital malformations and prematurity [5].

In the recent past, LMICs underwent a series of rapid changes in dietary consumption and physical activity. Traditional foods were replaced by processed foodstuffs leading to hypercaloric, high-fat diets. This shift became known as the nutrition transition, a term first proposed in 1993 by Barry Popkin [6] to reflect the recent, rapid changes in feeding and food patterns, which in the past history of mankind took thousands of years [7]. Global analyses show that mean BMI in women increased from 22.1 to 24.4 kg/m^2^ from 1975 to 2014, while underweight prevalence declined from 14.6 to 9.7% [8].

Country-level analyses show that national income is negatively associated with underweight and positively associated with the prevalence of overweight or obesity [9], but within-country social patterns of inequality vary according to national income levels. In 2004, Monteiro et al. showed that while there are strong positive associations between obesity and education in low-income countries, in high-income countries this pattern is reversed [10]. Several multicountry analyses in LMICs confirm the higher prevalence of overweight or obesity in the wealthiest quintile of women [11–13] but there is evidence that in the wealthier middle-income countries prevalence among the wealthiest women is lower than that in the intermediate wealth groups [12, 14]. Few multicountry analyses are available for high-income countries, of which the most informative is an analysis of World Health Surveys in 70 countries, including 31 high-income and 39 LMICs. This study did not separate the results by sex, but it confirmed that mean BMI increased with wealth in the poorest countries. As national prevalence of high BMI increased, mean BMI in the richest quintile fell below those in the other quintiles [12]. This finding is compatible with an analysis of 39 LMICs with at least two surveys from 1991 to 2008, which showed that prevalence is increasing more rapidly in the poorest than in the richest quintiles [15].

Therefore, the literature suggests that in low-income countries, overweight or obesity are the most prevalent among the wealthiest women. As countries become richer, this pattern is eventually inverted, leading to higher prevalence among poor women as is currently observed in high-income countries.

In contrast to the ample literature on socioeconomic inequalities and trends in overweight and obesity among women from LMICs, there are few studies of underweight. An analysis in 39 countries showed an inverse association between the prevalence of BMI < 16 kg/m^2^ and women’s education [16].

Our present analyses cover both ends of the malnutrition spectrum in women—underweight and overweight or obesity—in a larger number of LMICs than available in earlier studies. In addition to describing the magnitude of socioeconomic inequalities using summary indices, we report on the inequality pattern index, that is, on whether such inequalities are being primarily driven by the poorest or richest groups of women [17].

## Methods

We analyzed data from nationally representative surveys from 23 low-income and 26 middle-income countries according with the World Bank classification [18]. These surveys were carried out from 2010 and 2016 and their datasets are publicly available at the Demographic and Health Survey (DHS) program. Other types of national surveys, such as Multiple Indicator Cluster Surveys (MICS) [19] and Reproductive Health Surveys were considered, but MICS surveys could not be included due to the lack of anthropometric measures on women and RHS survey due to the lack of country reports after year 2008. The 2016 DHS from Timor-Leste was not included in the analyses as the quality of anthropometric data was questionable (UNICEF personal communication). In 28 of the 49 surveys, weight and height measurements were carried out in subsamples of women, using different sampling fractions in different surveys, and resulting in smaller sample sizes. Nevertheless, the selection of subsamples was intended to result in comparable sets of women with those included in the full sample. Sample sizes for the anthropometric analyses ranged from 2446 women aged 20–49 years for Lesotho 2014 to 531,443 in India 2015, with a median of 5891 women.

The DHS sampling design relies on multistage sampling to ensure national representativity. Standardized questionnaires are used to collect information from all women aged 15–49 years living in the sampled households. Anthropometric measurements were realized by trained field workers. Women weighed using digital scales without shoes and wearing light clothes and height was also measured without shoes with adjustable measuring boards [20]. Data collection at country level is under the responsibility of national agencies such as governmental statistical offices or non-governmental institutions, under the supervision of the DHS team. More detailed information about the DHS program methodology is available elsewhere [21].

Our population of interest was women aged 20–49 years, who were not pregnant nor had delivered in the last month before the survey. Adolescents were excluded as the fixed cutoff points for overweight and obesity for adult women are not recommended for this age group.

BMI was calculated by dividing weight in kilograms by the square of height in meters and classified into three groups using internationally agreed-upon cutoff points: underweight (<18.5 kg/m^2^), overweight (25.0–29.9 kg/m^2^), and obesity (≥30.0 kg/m^2^). In some analyses we combined the overweight and obese women (BMI ≥ 25.0 kg/m^2^) [22].

All surveys selected for analysis had available data on the wealth index in the original dataset. The index is estimated through principal component analysis, considering several household assets, building materials of the dwelling, and utility services such as water, sanitation, and electricity [23]. Because relevant assets may vary in urban and rural households, separate principal component analyses are carried out in each area, which are later combined into a single score using a scaling procedure to allow comparability between urban and rural households [24]. The index is divided into deciles, with ~10% of the households, with the first decile (D1) including the poorest 10% and the tenth decile (D10) the wealthiest 10% of all households in the sample.

We used per capita gross national income (GNI) data at constant purchasing power parity (in 2011 international dollars) from the World bank international comparison program database [25].

We calculated the prevalence of the three BMI outcomes across countries and within-countries, according to wealth deciles. T-tests were used to compare prevalence according to country income groups. Pearson correlation was used to analyze the relation between GNI per capita and underweight, overweight or obesity, and obesity. Fractional polynomials were used to predict prevalence of the outcomes according to per capita national incomes using the fracpoly Stata command. Absolute wealth-related inequalities were studied using the slope inequality index, derived from a logistic regression equation that takes into account the whole distribution of the outcome according to wealth [26]. The index is the slope of the resulting regression line and, represents the absolute difference—expressed as percent points—between the fitted value of the outcome in the wealthiest and poorest ends of the socioeconomic scale [26]. The index ranges from −100 to +100. A value of zero indicates that socioeconomic inequalities do not exist; positive values show higher prevalence of the outcome among rich women, and negative values indicate that the outcome is more common among the poor [27].

$${\mathrm{Inequality}}\,{\mathrm{pattern}}\,{\mathrm{index}} \,=\, \left( {{\mathrm{P}}_{{\mathrm{Q5}}}\,-\,{\mathrm{P}}_{\mathrm{n}}} \right)-\left( {{\mathrm{P}}_{\mathrm{n}}\,-\,{\mathrm{P}}_{{\mathrm{Q1}}}} \right) \,=\, {\mathrm{P}}_{{\mathrm{Q1}}} \,+\, {\mathrm{P}}_{{\mathrm{Q}}5}\,-\,2{\mathrm{P}}_{\mathrm{n}},$$

We also calculated the inequality pattern index. This summary measure assesses whether inequality is mainly driven by the richest individuals being well ahead of the rest of the population (“top inequality”) or by the poorest lagging behind the rest (“bottom inequality”). As currently proposed, the index relies on prevalence by wealth quintiles (rather than deciles). It is expressed in percent points, and calculated according to the formula below, where PQ1 stands for prevalence in the poorest quintile, PQ5 prevalence in the richest quintile, and PN the national prevalence [17].

All analyses were carried out with Stata version 15 (StataCorp. 2017. Stata Statistical Software: Release 15. College Station, TX: StataCorp LLC) taking into account the multi-stage, clustered nature of the sample design. Ethical clearance for data collection was obtained by the national institutions involved in data collection at country level. All analyses are based on anonymized, publicly available datasets.

## Results

Results by country are presented in Supplementary Table 1, which also shows the values of the inequality indices. In the text below, we refer the readers to the main figures. The supplementary materials provide more detailed results.

The median prevalence of underweight was 7.3% (ranging from 0.2% in Egypt to 20.5% in Ethiopia). For overweight or obesity, the median was 31.5% (ranging from 8.8% in Ethiopia to 85.3 in Egypt) and for obesity 10.2% (ranging from 1.9% in Ethiopia to 48.8 in Egypt).

**Fig. 1:**
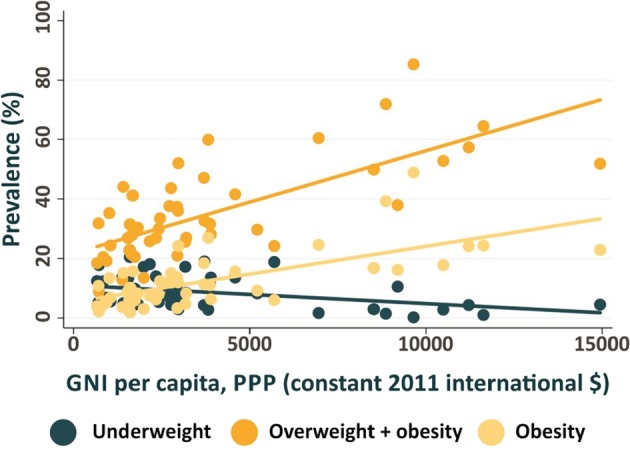
Prevalence of underweight, overweight or obesity, and overweight in 49 countries according to log mean national income per capita, 2010–2016.

Figure 1 shows that underweight prevalence tends to decline as GDP per capita increases, whereas the opposite is observed for overweight or obesity, and for obesity prevalence. The linear models fitted the data well, and there was no evidence of non-linearity as fractional polynomials were tested but failed to improve the fit. The Pearson correlation coefficients with log GNI were −0.33 for underweight, 0.72 for overweight or obesity, and 0.66 for obesity. The linear models explained 13.2% of the variability of underweight (*p* = 0.006), 50.0% for overweight or obesity (*p* < 0.001), and 44.1% for obesity (*p* < 0.001). The regression parameters are provided in Supplementary Table 2. The inverse association between the national prevalence of underweight with that of overweight or obesity is shown in Supplementary Fig. 1.

**Fig. 2:**
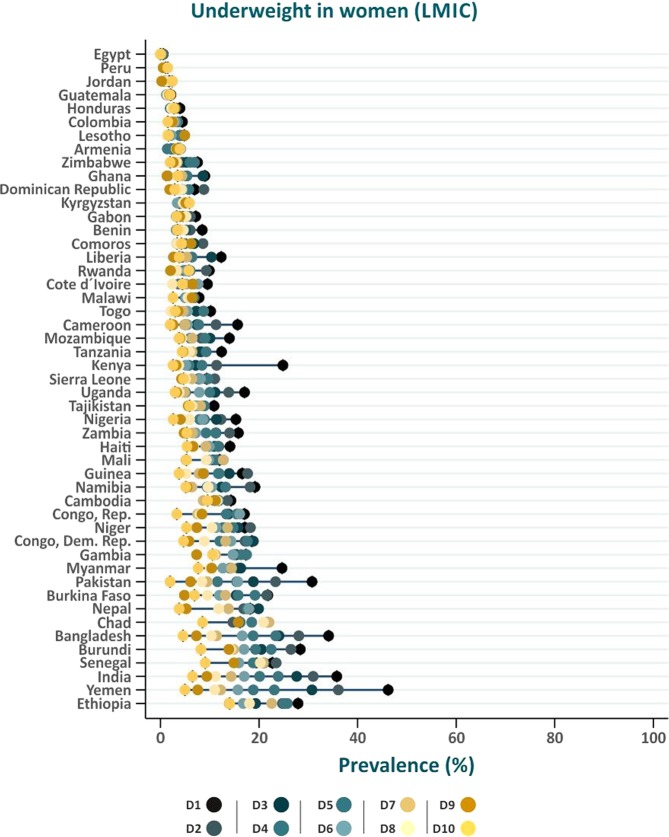
Prevalence of underweight by wealth deciles. Countries are ordered according to national prevalence.

Figure 2 shows socioeconomic inequalities in underweight prevalence according to wealth deciles. Countries with low prevalence do not show important inequalities, whereas high-prevalence countries tend to show that levels are inversely related to wealth. The slope index of inequality was negative in 45 countries of which 38 were significant, indicating higher prevalence among the poor than the rich. Only four countries (Armenia, Jordan, Kyrgyzstan, and Peru) had positive values, all with small magnitudes, ranging from 0.2 to 1.3% points. Analyses of inequality patterns show that in most countries the disparities are not being driven by one or two specific deciles. The exceptions are countries with inequality pattern indices above 4.0 (Cameroon, Kenya, Pakistan, Uganda, and Yemen) where the poorest deciles tend to show substantially higher prevalence than the rest of the population. On the other extreme, Chad and Nepal had substantially lower prevalence in the richest deciles than in the remaining deciles, with negative values (−7.7 and −6.1, respectively) for the inequality pattern index (Supplementary Table 1). These patterns may be observed in Fig. 2.

**Fig. 3:**
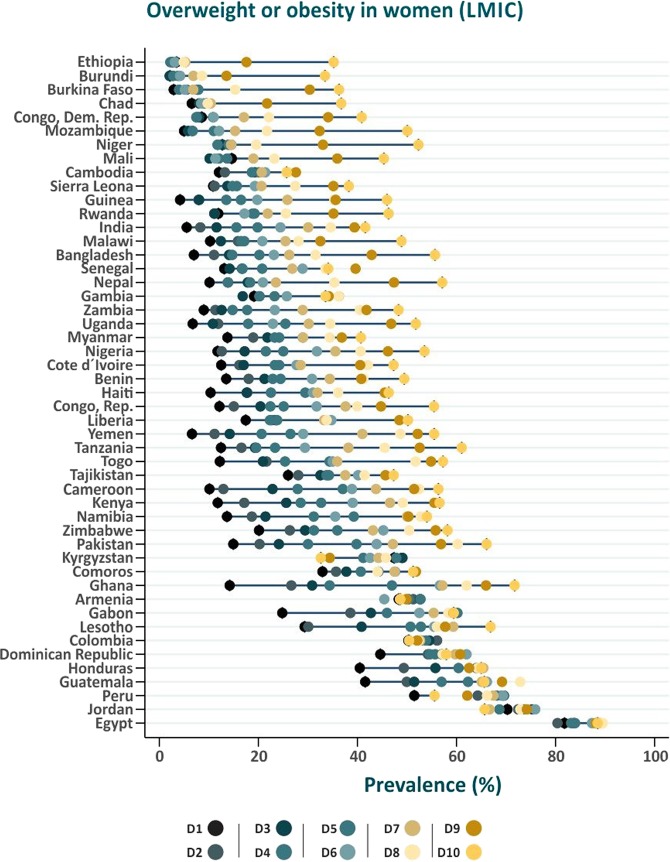
Prevalence of overweight or obesity by wealth deciles. Countries are ordered according to national prevalence.

Inequalities in the prevalence of overweight or obesity (Fig. 3) were much more marked than those observed for underweight. The SII was positive in 44 countries, all of them were significant. In most countries, inequalities are driven by prevalence in the wealthiest deciles, particularly in countries with low national prevalence. The inequality pattern index was >10% points in eight countries, seven of which are in Sub-Saharan Africa (Burkina Faso, Burundi, Chad, DR Congo, Mali, Mozambique, Nepal, and Niger). The opposite trend, that is, inequality being driven by markedly lower prevalence among the poorest women, was found in high-prevalence countries such as Honduras, Lesotho, Gabon, Peru, and Ghana, all of which had inequality pattern indices below −10% points.

Results for obesity by wealth deciles are shown in Supplementary Fig. 2. The observed patterns are similar to those presented in Fig. 3, although prevalence levels are lower. In many countries, obesity prevalence was noticeably higher in the wealthiest than in the second wealthiest decile.

**Fig. 4:**
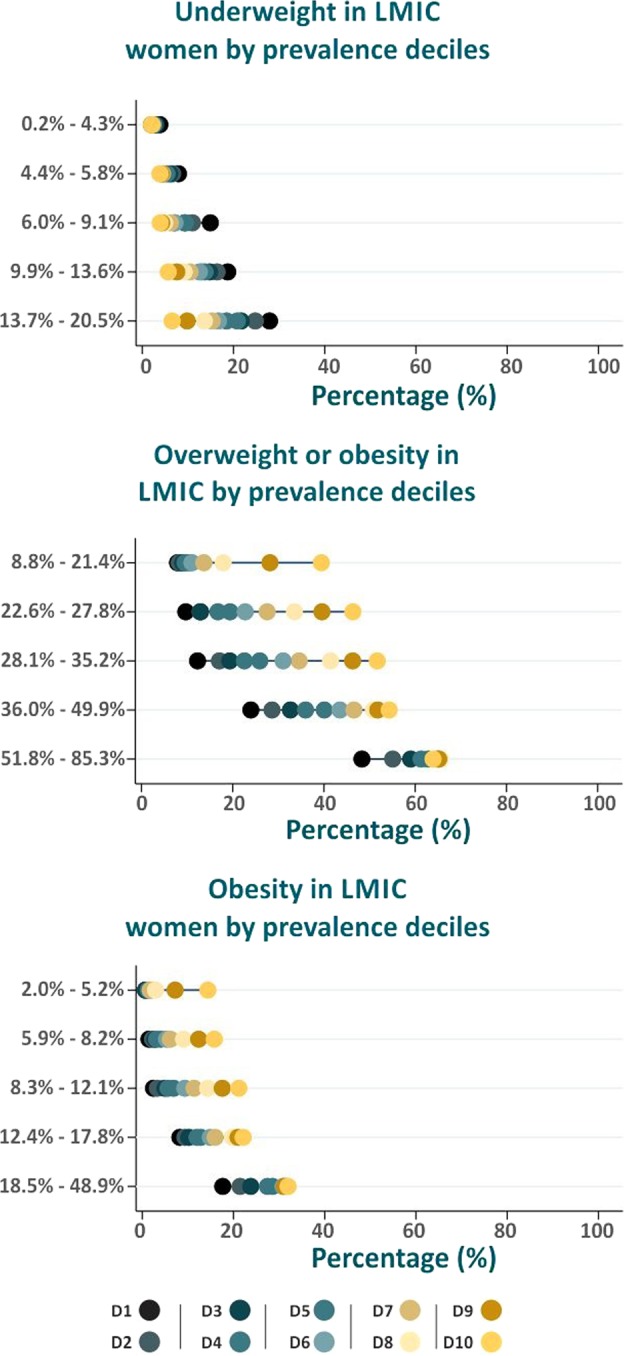
Mean prevalence of underweight, overweight or obesity and obesity by wealth deciles. Average values of countries in different prevalence strata.

The above results suggest that national prevalence drives the patterns of inequality. This hypothesis was tested in Fig. 4. Countries with low prevalence of underweight show low levels in all wealth deciles, but as national prevalence levels increase, the wealthiest deciles tend to be spared. Values of the slope and inequality pattern indices are shown in Supplementary Table 3. The slope index increases with prevalence, whereas the inequality pattern index does not show any clear trend.

In contrast, the analyses of overweight or obesity show that in low-prevalence countries, the richest women tend to show levels that are well above the rest. At high-prevalence levels, although the poorest women remain slightly behind the other deciles, overweight, and obesity affect the whole population. According to the slope index, the widest absolute inequalities are present in the intermediate prevalence groups (Supplementary Table 3). The pattern index is positive when prevalence is low and negative at the higher prevalence levels.

## Discussion

Our country-level analyses confirm the existing literature [14, 28], by showing that national income levels are negatively associated with underweight and positively associated with overweight or obesity. Differently from the literature, we show that the associations with the overweight or obesity are not linear, reflecting increased slopes of the curve at higher prevalence levels.

Our analyses of within-country socioeconomic inequalities for women were based on 49 countries, a larger number than were included in earlier analyses that reported on a maximum of 39 countries. The literature on inequalities in women’s underweight is even more limited [16, 29, 30], but it consistently shows higher prevalence among poor women as was found in the present analyses where in 45 of the 49 countries the slope index was negative. Although some countries (Fig. 2) show prevalence levels in the poorest deciles that are well above those in the rest of the population, this was not observed for most countries. The inequality pattern index did not vary substantially with national prevalence.

Regarding overweight or obesity, 44 of the 49 countries had positive values for the slope index, indicating that prevalence increased with wealth. The smallest magnitudes of the slope index were observed for high-prevalence countries, thus suggesting that the gap between poor and rich women is closing down in high-prevalence countries. This finding is consistent with the literature summarized in the introduction [9, 11–13]. Inequality patterns varied markedly with national prevalence. In low-prevalence countries, “top inequality” was present, that is, rich women have prevalence levels well above the rest of the population, whereas in high-prevalence countries the pattern corresponds to “bottom inequality”, observed when poor women have substantially lower prevalence than the other deciles. The results for obesity were similar to those obtained for the combined outcome of overweight or obesity. Our analyses, based on wealth deciles, are more revealing of high-risk subgroups than the more traditional division by quintiles used in the literature so far.

Because our analyses were restricted to LMICs, we were unable to confirm the finding from other studies—such as that by Monteiro et al. [10]—showing higher prevalence of overweight or obesity in poor, less educated than in rich, more educated women. Nevertheless, suggestive patterns that this is taking place were observed in some upper-middle-income countries such as Colombia, Kyrgyzstan, Peru, and Jordan.

Our analyses have several strengths. These include covering a larger number of countries than previous analyses, using measured weight and heights rather than reported values as in other studies [12], relying on nationally representative sample surveys, using a standardized measure of socioeconomic position that results in equal-sized groups, and analyses by wealth deciles. Lastly, this is the first set of analyses to report not only on the magnitude of inequalities but also on inequality patterns.

Several limitations must be acknowledged. As mentioned, over half of the surveys included anthropometric measures on subsamples of all women; yet, among women in the subsamples, missing values were uncommon with a median of 1.0%, and all datasets had 90% or more nonmissing values.

Another limitation is the time-lapse between the surveys; although the analyses were limited to countries with a survey in 2010 or later, 18% of the surveys had data from before 2012, and it is possible that with rapid transition these prevalence levels are no longer valid. Data were available for a higher proportion of all low-income countries (46.9%) than for lower-middle (42.9%) or upper-middle (10.2%) income countries. Due to the lack of DHS surveys in large upper-middle-income countries (such as Brazil, South Africa, China, Mexico, etc.) and in high-income countries, it was not possible to test the hypothesis that in these countries the social gradient in overweight and obesity would have been reversed. In fact, our results for high-prevalence countries (Figs. 3 and 4) show a narrowing of inequalities, rather than a reversal. Finally, measurement of socioeconomic position using asset indices has its limitations [28]. Nevertheless, urban or rural residence was taken into account when calculating the index [24], and the clear gradients observed in our analyses suggest that asset indices are able to discriminate among different subgroups of the population.

Our results may be interpreted in light of four of the five stages of the nutrition transition [31], given that the first stage (hunter-gatherer societies) is extremely rare in present-day societies. The second (modern agriculture and famine) and third (receding famine as incomes grow) stages may be applicable to the poorest women in low-income countries, among whom underweight prevalence is substantial. The high prevalence of overweight and obesity observed in middle-income countries, particularly among better-off women, reflects the fourth stage of the transition (degenerative disease due to changes in activity levels and diet). Finally, we detected patterns in a few upper-middle-income countries (Colombia, Kyrgyzstan, Peru, and Jordan) suggesting that women in the richest deciles may be moving into the fifth and last stage of the transition (behavioral change in which populations reduce their fat, increase fiber intake, and do meaningful physical activity). Our disaggregated analyses show that, far from being applicable to whole countries, the stages of the nutrition transition affect women from different social classes to different extents, signaling that within the same country several stages of the transition may be observed. In Pakistan, for example, the prevalence of underweight in the poorest decile is 31% whereas the prevalence of overweight or obesity in the richest decile reaches 66%.

Our results have policy implications. Underweight is affecting specific groups of very poor women in very poor countries. Targeting interventions at these groups is essential, given that even in most low-income countries the majority of women are not affected. Effective interventions may range from food and micronutrient supplementation to well as intersectoral anti-poverty and women’s empowerment initiatives [32].

Regarding the prevention of overweight and obesity, analyses from high-income countries show that the development of new technologies in food processing, allied with urbanization, have led women to favor processed foods, instead of buying fresh ingredients for cooking at home, and this shift has contributed to the epidemic of overweight and obesity [33]. Our analyses of inequality patterns show that overweight is being driven by the wealthier women, particularly in the poorest countries. Given that rich women in these countries tend to be early adopters [34] of western patterns of diet and physical activity, preventive strategies should be targeted at these groups of women who are likely to act as trend-setters [17] in the early stages of the overweight epidemic.

Finally, overweight and obesity in middle-income countries are trickling down, from wealthier women to the rest of the population, thus requiring population-level approaches such as food regulation, labeling, and taxation [35, 36], and well as environmental and behavioral interventions to promote physical activity, although the evidence supporting dietary approaches is far greater than that for the promotion of exercise [37].

Since specific population subgroups within a given country may be at distinct stages of the nutrition transition and may also show different responses to nutrition interventions, it is essential to gather regular information that may be disaggregated by population subgroups, such as wealth quintiles or deciles. Nutrition monitoring with an equity lens should become an integral component of tracking progress towards optimal nutrition at population level.

## Supplementary information

Supplementary figure 1

Supplementary figure 2

Supplementary table 1

Supplementary table 2

Supplementary table 3
