# Supplementary figures and images for: Socioeconomic inequalities in the prevalence of underweight, overweight, and obesity among women aged 20–49 in low- and middle-income countries
Source: Int J Obes (Lond). 2019 Dec 18;44(3):609–16. doi: 10.1038/s41366-019-0503-0 (PMC7046525; doi:10.1038/s41366-019-0503-0)

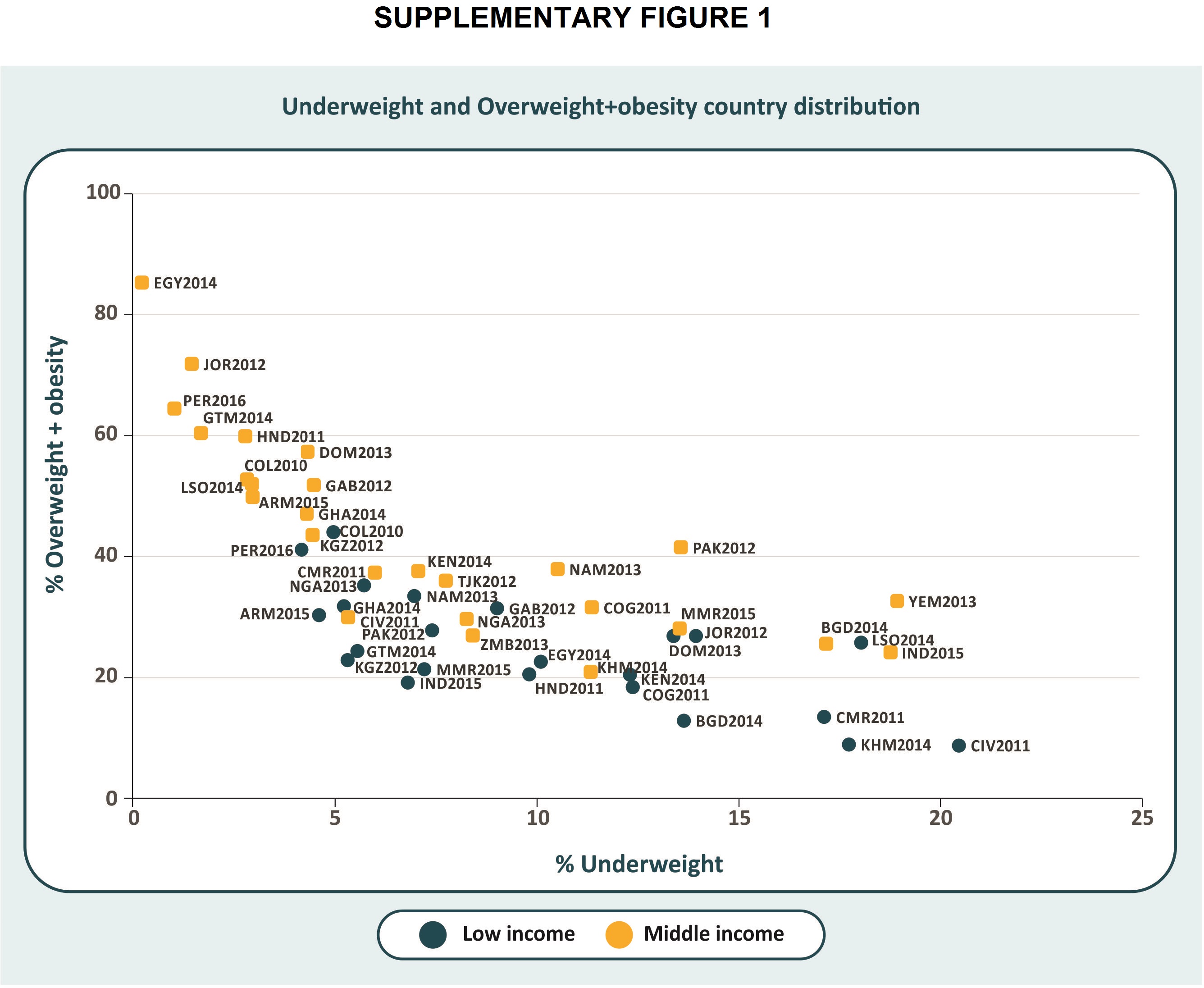

Supplement: Supplementary file 1 — Supplementary figure 1 [file 41366_2019_503_MOESM1_ESM.jpg]

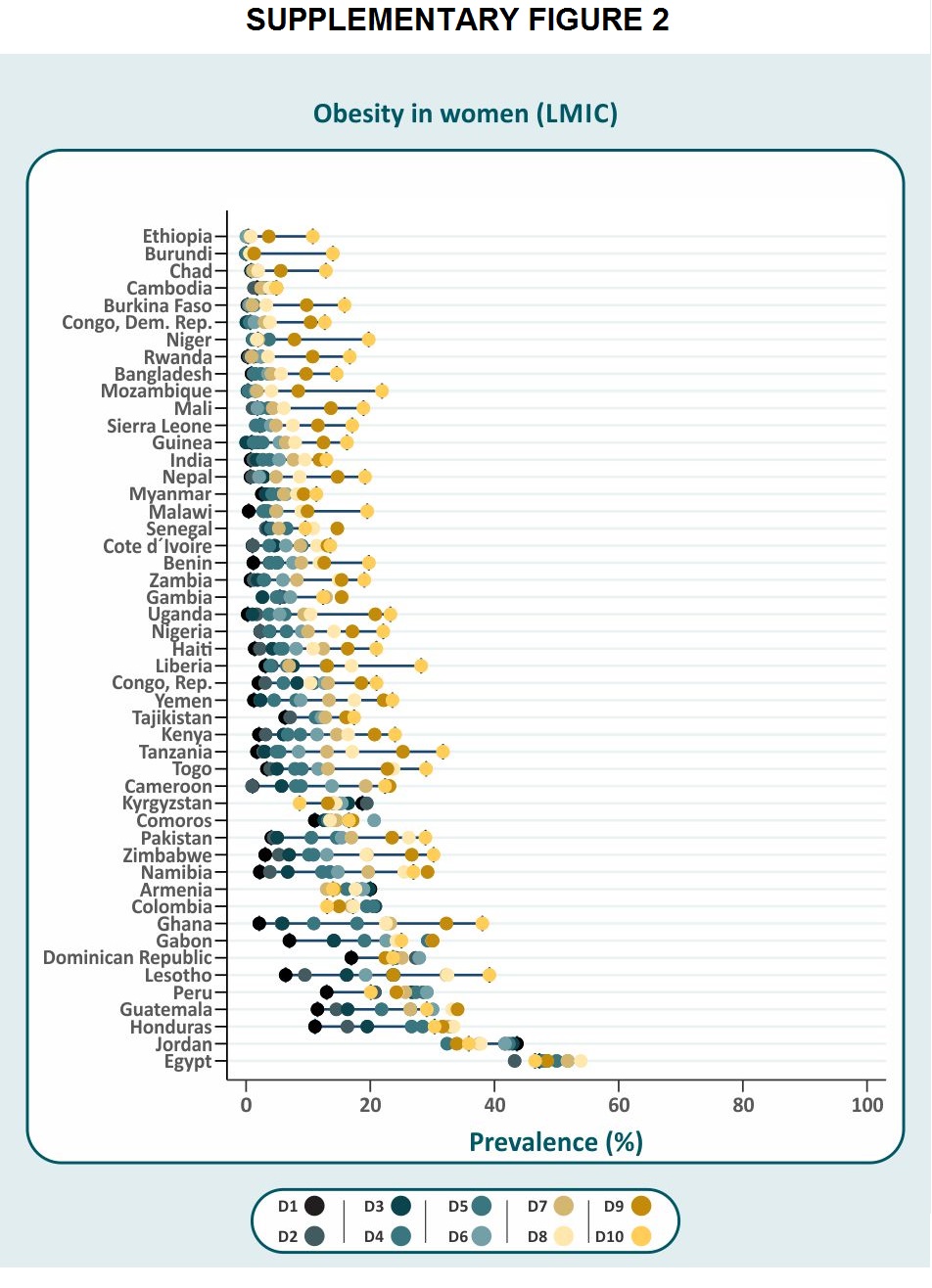

Supplement: Supplementary file 2 — Supplementary figure 2 [file 41366_2019_503_MOESM2_ESM.jpg]
